# HPV-16-Induced Squamous Cell Carcinoma in Hidradenitis Suppurativa: HPV Vaccination May Be Useful

**DOI:** 10.3390/cancers17040702

**Published:** 2025-02-19

**Authors:** Nessr Abu Rached, Riina Käpynen, Martin Doerler, Lennart Ocker, Carolin Frost, Yannik Haven, Falk G. Bechara

**Affiliations:** 1 International Centre for Hidradenitis Suppurativa/Acne Inversa (ICH), Department of Dermatology, Venereology and Allergology, Ruhr-University Bochum, 44791 Bochum, Germany; riina.kaepynen@kklbo.de (R.K.); martin.doerler@kklbo.de (M.D.); lennart.ocker@kklbo.de (L.O.); yannik.haven@kklbo.de (Y.H.); falk.bechara@kklbo.de (F.G.B.); 2 Skin Cancer Center, Department of Dermatology, Venereology and Allergology, Ruhr-University Bochum, 44791 Bochum, Germany

**Keywords:** hidradenitis suppurativa (HS), acne inversa, malignoma, squamous cell carcinoma, HPV

## Abstract

Introduction: Hidradenitis suppurativa (HS) is a chronic inflammatory skin disease associated with significant morbidity. Although cutaneous squamous cell carcinoma (cSCC) is a rare complication, it has serious consequences, particularly when associated with high-risk human papillomavirus (HPV) infections. This study examines two cases of HPV-16-induced cSCC in patients with long-standing HS and explores the potential role of HPV vaccination in preventing such malignancies. Methods and results: We report on two male patients with severe HS (Hurley stage III) and cutaneous squamous cell carcinoma with positive detection of HPV DNA in the tumour tissue. Conclusions: HPV vaccination may offer a preventive approach to cSCC in HS patients by reducing high-risk HPV infections. Incorporating vaccination into the management of HS, particularly in high-risk individuals, could potentially reduce the incidence of malignant transformation and improve long-term outcomes. Further research is warranted to validate these findings and refine prevention strategies.

## Simple Summary

Chronic lesions of hidradenitis suppurativa (HS) may rarely be associated with HPV-16-induced cutaneous squamous cell carcinoma (cSCC), which carries a high risk of severe progression. The results of this study highlight that HPV vaccination could be a preventive strategy to reduce the risk of such malignancies in HS patients.

## 1. Introduction

Hidradenitis suppurativa (HS) is an autoimmune chronic inflammatory disease that can lead to recurrent nodules, abscesses, scarring and fistulae. Cutaneous squamous cell carcinoma (cSCC) is an uncommon complication of HS [1]. Usually, cSCCs develop a long time after initial diagnosis of HS, probably as a result of repeated phases of extensive inflammation [2]. The cSCCs on HS lesions show a fulminant course with fatal outcome in many patients and requires a thorough physical examination of the skin lesions [3]. We present two cases of human papillomavirus (HPV)-16 induced HS-associated cSCC in two men with long histories of high inflammatory severe HS, smoking history and previous or ongoing adalimumab therapy. The benefit of HPV vaccination in HS patients is still unknown. HPV vaccination can reduce the risk of various cancers. We postulate that high-risk HS patients should definitely also be vaccinated against HPV for cSCC.

## 2. Case 1

A 59-year-old man with a 40-year history of HS presented to our clinic with severe and therapy-refractory HS. As multiple regimens of oral antibiotics and incisional drainage of the fistula tracts were ineffective, his primary care physicians initiated biologic therapy with adalimumab. The patient had a smoking history of over 30 pack-years. He was initially treated at his local hospital in a urology department due to the current deterioration of the clinical picture of genital HS and his general condition for sepsis and local infection. The patient was finally transferred to our centre for further treatment.

**Figure 1:**
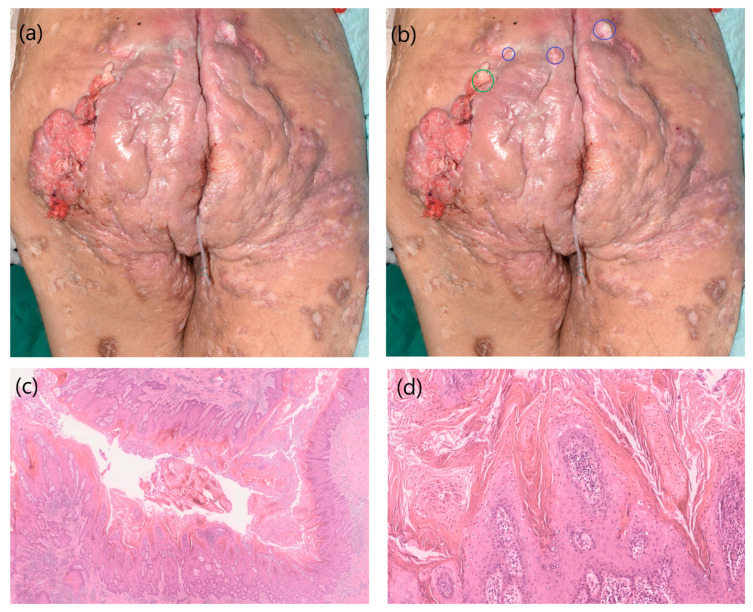

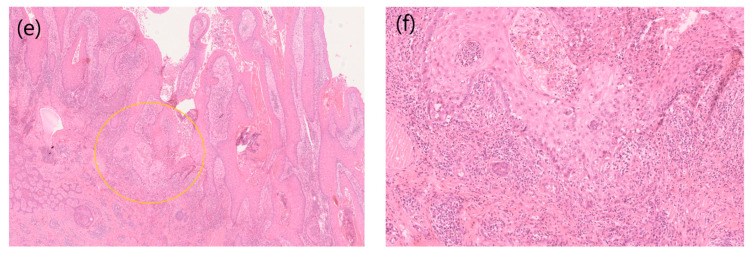
(**a**) The photo shows the perianal and gluteal area of the HS patient at first presentation. There is massive inflammation with lymphoedema and several verrucous tumours; (**b**) the photo shows the verrucous tumour with detection of high-risk type HPV-16 by PCR (circled in green) and the benign viral papilloma with detection of low-risk type HPV-6 by PCR; (**c**) histopathological examination of the blue circled area shows a benign papillomatous tumour located in the area of the fistula tract (HE staining); (**d**) characteristics of viral induction (parahyperkeratosis projected onto the papilla tips and coarsened keratin granules and perinuclear vacuole formation in the stratum granulosum) are visible at 100× magnification (HE stain); (**e**) cutaneous squamous cell carcinoma is visible in the yellow circled area (HE stain); (**f**) infiltrative-growing epithelial nests and strands with pleomorphic tumour cells are visible at 100× magnification (HE stain).

On arrival, clinical examination revealed severe HS (Hurley stage III) of the anogenital, gluteal, axillary and thigh regions with marked inflammatory lesions, fistula tracts, inflammatory nodules and gluteal ulcerations with atypical fungating plaques and surrounding induration (Figure 1a,b). On presentation to our department, multiple deep biopsies were taken for histological analysis due to the atypical clinical presentation of the gluteal HS lesion. Biopsies in the green highlighted area revealed a cutaneous squamous cell carcinoma that had developed in the area of the HS fistula tract (Figure 1a,b,e,f). High-risk HPV-16 was detected in the cSCC area. In the blue area, there was only a benign viral papilloma (Figure 1c,d). Interestingly, only the low-risk HPV-6 type was detected by polymerase chain reaction (PCR) in this area.

Further imaging showed enlarged reactive inguinal lymph nodes, but no metastases. The tumour was inoperable as the surgical margins of the cSCC could not be defined. A combination of anti-inflammatory therapy with secukinumab and anti-tumour therapy with cemiplimab was initiated.

## 3. Case 2

The second patient, a 54-year-old man with medical history of over 30 years of severe HS, regularly treated at our department, presented after several months abroad due to increased inflammatory activity of HS under ongoing adalimumab-therapy. The therapy regime of adalimumab 80 mg subcutaneously every 2 weeks was started in September 2019 and showed good effect with reduction in inflammatory activity, pain and fistula drainage for the past years. To the current moment, the patient is a non-smoker but reports an over 30 pack-year smoking history. Clinical examination revealed highly inflammatory fistulas and nodes in the pubic region, genitals, anal, perineum and buttocks. The perineum showed atypical hypergranulated lesions.

Similar to the first case, multiple deep biopsies for histological analysis were performed due to the atypical clinical appearance. Tissue biopsy revealed cSCC. HPV-16 was also found to be present in the tumour area of the cSCC. Fortunately, further imaging showed no evidence of metastases but extensive pelvic HS invasion. After a detailed discussion of the findings and treatment options, the patient expressly decided against radical surgery. Drug-based tumour therapy with cemiplimab combined with anti-inflammatory therapy of the HS with Bimekizumab was initiated. To date, the therapy has been well tolerated by both patients, although the further course is still pending..

## 4. Discussion

All therapy-resistant atypical nodules and plaques should be monitored and investigated in HS patients. HS-associated cSCC tends to involve the lower body (especially perianal/perineal) and may be associated with HPV. In addition, ongoing anti-TNF therapy may increase the risk and should be monitored to exclude malignant transformation, especially in high-risk patients. Histologically, the diagnosis of cSCC in HS lesions can be complicated by the high level of inflammation and benign, papillotomous tumour and requires careful macroscopic and microscopic examination.

In most cases, wide surgical excision offers a near definitive intervention and should at least be considered in all chronic HS patients due to the high morbidity and risk of malignant transformation. However, cemiplimab, an antibody immunotherapy that inhibits programmed cell death protein-1 and is approved for the treatment of locally advanced or metastatic squamous cell carcinoma, should be considered in patients ineligible for curative surgery and/or radiotherapy. Our two cases show that high-risk HPV type 16 infection can lead to malignancy in cSCC on HS lesions. Further research is needed in the area of prevention of SCC in HS patients, but HPV vaccination may be a useful strategy to prevent high-risk human papillomavirus (HR-HPV)-associated SCCs in these patients.

The majority of cSCCs are located on sun-exposed areas, indicating UV radiation as the main oncogenic factor [4]. Infection with HPV was also reported to be associated with cSCC development, in particular in immunosuppressed patients [5,6]. HPV types detected in skin cancers mainly belong to the genus beta HPV [7,8]. Their role in cancer development, however, differs from those of alpha HPVs in genital cancers. While the presence and constant expression of oncogenes of high-risk (HR) alpha HPV is required for the initiation and maintenance of the cancer phenotype, beta HPV appears to only be involved in the early stages of skin cancer development, but is no longer required to maintain the malignant phenotype [9,10]. Although the predominating HPV types detected in cSCC belong to the genus beta HPV, some SCC and in situ forms, i.e., periungual and digital SCC as well as extragenital Bowen disease, are mainly associated with mucosal alpha HPV types [11].

Immunization against high-risk HPV using L1-virus-like particles (VLPs) was shown to be very effective in preventing HPV infection and the subsequent development of anogenital tumours [12,13]. L1 represents the major capsid protein and currently licensed vaccines cover HPV 16 and 18 (Cervarix), HPV 6, 11, 16, and 18 (Gardasil), and HPV 6, 11, 16, 18, 31, 33, 45, 52, and 58 (Gardasil-9), respectively. Since the efficacy of VLP-based vaccines is based mainly on the induction of neutralizing antibodies preventing de novo HPV infection [12,14], they were not recommended to treat existing HPV infections [15]. Therapeutic vaccines targeting E6 and E7 proteins, which are constantly expressed in infected cells, are probably more promising for the treatment of established HPV infection.

Nevertheless, the licensed HPV L1-VLP-based vaccines have been used to manage different lesions with active or pre-existent HPV infection. Successful treatment of genital and cutaneous warts with these vaccines has been reported in several studies [16,17,18,19,20,21,22]. For instance, in a study comparing standard therapy (cryotherapy, nitrizinc complex, Imiqimod 5% or photodynamic therapy) and combined therapy (standard therapy plus vaccination with Gardasil-9) for treatment of anogenital and oral warts, the clinical response rate (complete or partial clearance) was significantly higher in patients receiving the combined therapy [18].

Genital warts are caused mainly by HPV types 6 and 11 and result from productive HPV infection. The efficacy of vaccination with L1-VLPs may relate to the neutralization of newly formed virus particles by vaccine-induced anti L1-antibodies that prevent the spreading of HPV infection to adjacent cells of the mucosal epithelium.

In contrast, in anogenital cancers, HR alpha HPV is often integrated into the host genome and virus particles are no longer produced [23]. Thus, the therapeutic efficacy of L1-VLP-based vaccines appears to be questionable in HPV-associated cancers, although non-specific immune stimulation induced by the adjuvants in the vaccine formulations may confer beneficial effects.

Due to the prevention of primary infection, vaccination with HPV L1 VLPs should be carried out before first sexual contacts and thus is generally recommended in young girls and boys from the age of 9 years. The age range for vaccination varies in different national immunization programs and is between 9 and 25 years in most European countries [24]. Individuals older than 25 years of age may also be vaccinated, in particular when they are at increased risk for HPV infection, such as for instance men who have sex with men (MSM) [25]. Moreover, application of HPV vaccination in patients with cervical and anal high-grade squamous intraepithelial lesions (HSILs) after surgical treatment reduces the risk of recurrent disease and potentially the development of cervical and anal cancer [26,27,28,29]. The mechanisms behind the beneficial effect of the post treatment HPV vaccination are still unclear, but may relate to the stimulation of local immune responses that block the entry of newly formed virus particles into uninfected cells of the epithelial basal layer. Furthermore, the adjuvant components of the vaccines may improve T cell immunity against HPV oncogenes, which, in patients with cervical dysplasia, is frequently impaired and insufficient to control HR HPV infection [30,31].

Patients with long-standing HS disease and chronic inflammation may represent another risk group for the development of anogenital SCC. Although which HPV types dominate in HS-associated SCC has not yet been thoroughly studied, HR alpha HPVs seem to be involved most frequently [32]. The two patients in our study were also shown to harbour HR HPV type 16 in SCC tissue samples. In another study, HPV was not detected in any of 12 anogenital SCCs from HS patients (gluteal, perianal, perineal and vaginal location); however, the failure of HPV detection may relate to the application of less sensitive HPV detection methods [33]. Based on serological data, a substantial proportion of the population has obviously never had contact with these viruses [34,35]. For this reason, HPV vaccination may be useful in HS patients.

## 5. Conclusions

In conclusion, HPV vaccination appears to be a reasonable procedure to prevent the development of HR HPV-associated anogenital cancer in HS patients. If not administered in childhood, HPV vaccination should be performed in the early stages of HS. Even when patients are older than 26 years, vaccination can be beneficial, as infection with HR HPV does not always occur in adolescence.

## Data Availability

The data that support the findings of this study are available on request from the corresponding author. The data are not publicly available due to privacy or ethical restrictions. It is confirmed that all authors had access to the data and were involved in writing the manuscript.
